# Advancing the Fight Against Cervical Cancer: The Promise of Therapeutic HPV Vaccines

**DOI:** 10.3390/vaccines13010092

**Published:** 2025-01-19

**Authors:** Qian Zheng, Misi He, Zejia Mao, Yue Huang, Xiuying Li, Ling Long, Mingfang Guo, Dongling Zou

**Affiliations:** 1 Chongqing University Cancer Hospital, School of Medicine, Chongqing University, Chongqing 400030, China; zhengqian75@cqu.edu.cn (Q.Z.); 202237021083@stu.cqu.edu.cn (Z.M.); 15213380833@163.com (L.L.); 2 Department of Gynecologic Oncology, Chongqing University Cancer Hospital & Chongqing Cancer Institute & Chongqing Cancer Hospital, Chongqing 400030, China; hems1020@cqu.edu.cn (M.H.); huangyue@cqu.edu.cn (Y.H.); xiuyingli2017@cqu.edu.cn (X.L.); cq_cancerhospital@cqu.edu.cn (M.G.); 3 Chongqing Specialized Medical Research Center of Ovarian Cancer, Chongqing 400030, China; 4 Organoid Transformational Research Center, Chongqing Key Laboratory for the Mechanism and Intervention of Cancer Metastasis, Chongqing University Cancer Hospital, Chongqing 400030, China

**Keywords:** therapeutic vaccines, human papillomavirus, cervical cancer, cervical intraepithelial neoplasia

## Abstract

Human papillomavirus (HPV) is a major global health issue and is recognized as the leading cause of cervical cancer. While prophylactic vaccination programs have led to substantial reductions in both HPV infection rates and cervical cancer incidence, considerable burdens of HPV-related diseases persist, particularly in developing countries with inadequate vaccine coverage and uptake. The development of therapeutic vaccines for HPV represents an emerging strategy that has the potential to bolster the fight against cervical cancer. Unlike current prophylactic vaccines designed to prevent new infections, therapeutic vaccines aim to eradicate or treat existing HPV infections, as well as HPV-associated precancers and cancers. This review focuses on clinical studies involving therapeutic HPV vaccines for cervical cancer, specifically in three key areas: the treatment of cervical intraepithelial neoplasia; the treatment of cervical cancer in combination with or without chemotherapy, radiotherapy, or immune checkpoint inhibitors; and the role of prophylaxis following completion of treatment. Currently, there are no approved therapeutic HPV vaccines worldwide; however, active progress is being made in clinical research and development using multiple platforms such as peptides, proteins, DNA, RNA, bacterial vectors, viral vectors, and cell-based, each offering relative advantages and limitations for delivering HPV antigens and generating targeted immune responses. We outline preferred vaccine parameters, including indications, target populations, safety considerations, efficacy considerations, and immunization strategies. Lastly, we emphasize that therapeutic vaccines for HPV that are currently under development could be an important new tool in fighting against cervical cancer.

## 1. Introduction

Globally, cervical cancer (CC) is the fourth highest in incidence and mortality among female cancers [1]. The year 2020 recorded approximately 598,000 new cases and 338,800 deaths, constituting 6.5% and 7.7% of total new cancer cases and deaths in women, respectively [2]. The incidence and mortality rates of CC are significantly higher in countries with a low Human Development Index (HDI), with the incidence rate in low-HDI countries being three times that of high-HDI countries, and the mortality rate being six times higher [3]. Few diseases reflect global inequality as starkly as cervical cancer, primarily due to the low human papillomavirus (HPV) vaccination rates and lack of effective prevention and management measures for cervical cancer in low-HDI countries.

Persistent high-risk HPV infection is the leading cause of CC, with 70% of CC associated with HPV types 16 and/or 18 [4]. Vaccination is the most effective means of controlling the occurrence of CC. Currently, there are two types of HPV vaccines. One is the prophylactic HPV vaccine, which is mainly aimed at preventing new HPV infections and has been widely promoted. The other is the therapeutic HPV vaccine, which is still in clinical research and aims to eradicate or treat existing HPV infections, as well as precancerous lesions and cancers related to HPV. Although prophylactic HPV vaccination programs have significantly reduced HPV infection rates and the incidence of CC [5,6], the burden of CC remains considerable, especially in developing countries where vaccine coverage and vaccination rates are insufficient [7]. The development of therapeutic HPV vaccines is an emerging strategy that has the potential to enhance the prevention and control of CC.

This review offers a comprehensive overview of the existing therapeutic HPV vaccine landscape for treating cervical intraepithelial neoplasia (CIN) and CC. First, we describe the mechanisms of action and indications for both prophylactic and therapeutic HPV vaccines. Subsequently, we review the completed and ongoing clinical trials, summarizing the role of therapeutic HPV vaccines in high-risk HPV infections, CIN, and CC. Finally, we summarize the current characteristics and limitations of therapeutic HPV vaccine development and discuss future prospects for this emerging treatment modality.

## 2. Prophylactic HPV Vaccine

HPV is a double-stranded Deoxyribonucleic Acid (DNA) virus without an envelope, encoding eight proteins: the capsid proteins L1 and L2, and the early regulatory proteins E1, E2, E4, E5, E6, and E7 [8]. Prophylactic HPV vaccines function by employing virus-like particles (VLPs), composed of the HPV capsid proteins L1 and L2, as antigens to stimulate the humoral immune system into producing specific neutralizing antibodies, thereby preventing HPV infection. Clinical results show that the neutralizing antibodies generated by prophylactic HPV vaccines can penetrate the vascular wall to reach the site of infection, bind to the virus, and render it incapable of infecting cells [9]. This type of vaccine does not contain the genomic components of the virus, introduces no risk of carcinogenicity, and has a good safety.

Currently, there are five prophylactic HPV vaccines that have been both launched globally and prequalified by the World Health Organization (WHO). These include the following: (i) the quadrivalent vaccine (Gardasil^®^, targeting HPV types 6, 11, 16, and 18), launched in 2006 by Merck & Co., Inc., Kenilworth, NJ, USA; (ii) the bivalent vaccine (Cervarix™, targeting HPV types 16 and 18), launched in 2007 by GlaxoSmithKline, Brentford, UK; (iii) the nonavalent vaccine (Gardasil-9^®^, targeting HPV types 6, 11, 16, 18, 31, 33, 45, 52, and 58), launched in 2014 by Merck & Co., Inc., USA; (iv) the bivalent vaccine (Cecolin^®^, targeting HPV types 16 and 18), launched in 2019 by Xiamen Innovax Co. Ltd., Xiamen, China; (v) and the bivalent vaccine (Walrinvax^®^, targeting HPV types 16 and 18), launched in 2022 by Walvax Biotechnology Co. Ltd., Kunming, China [10].

Prophylactic HPV vaccines are primarily used in the following situations: (i) they are most effective in women who have not had sexual intercourse and have not been infected with HPV; (ii) it also has a good protective effect on women who have been previously infected with HPV [11]; (iii) even if currently infected with HPV, vaccination with the nine-valent prophylactic HPV vaccine can prevent infection with other vaccine-type HPV strains and related lesions [12]; (iv) after treatment for CIN caused by HPV, vaccination with the prophylactic HPV vaccine can also prevent HPV reinfection, thereby avoiding the recurrence of CIN [13]. After surgical removal of the lesion, the antibodies induced by the prophylactic HPV vaccine can prevent new infections from different types and reinfections from the same type [14].

WHO launched the “Global Strategy to Accelerate the Elimination of Cervical Cancer as a Public Health Problem” on 17 November 2020 [15], which was actively responded to by 194 member states, including China. The strategy aims to achieve an elimination standard of no more than four new cases of cervical cancer per 100,000 women by the next century. To realize this strategic goal, the 90-70-90 targets must be reached by 2030, which means 90% of girls should complete all doses of the prophylactic HPV vaccine before the age of 15. However, the current global strategy’s launch and implementation is significantly behind its 2030 targets. By 2020, the vaccination rate of the prophylactic HPV vaccine among adolescent girls worldwide was only 13% [16]. In some economically developed countries, such as Canada, the United Kingdom, and Australia, the vaccination rate has exceeded 80% [17]. In Australia, the prevalence of HPV has dropped from 22.7% to 1.1% [18].

However, due to low prophylactic vaccine coverage, there is a large population of individuals already infected with HPV in developing countries who cannot benefit from prophylactic vaccines and require therapeutic vaccines. Taking China as an example, all types of prophylactic HPV vaccines have been on the market in China for a short time, with the earliest bivalent vaccine Cervarix being launched in China in 2016 [19]. Due to various factors, such as the high cost of imported prophylactic HPV vaccines, limited supplies, late market launches, age requirements for vaccination, and insufficient awareness [7], many women have missed the best opportunity for vaccination (no sexual life, no contact with HPV). According to survey data from 2021 [7], only 3% of Chinese women have been vaccinated with the prophylactic HPV vaccine.

Despite the high efficacy of prophylactic HPV vaccines, the global vaccination rate is low, with significant regional disparities, and it cannot eliminate existing infections or treat CIN that has already occurred. On 3 July 2024, the WHO released the “WHO Preferred Product Characteristics for Therapeutic HPV Vaccines” report [20], which emphasizes the numerous challenges in the implementation of the current global strategy to eliminate cervical cancer, especially in low- and middle-income countries where cervical cancer screening and treatment services are limited. There is an urgent need to develop effective therapeutic HPV vaccines, illustrating the positive role of therapeutic HPV in the cervical cancer elimination strategy.

Therefore, the development of therapeutic HPV vaccines is another important means by which to achieve the WHO’s global strategy goals and is a strong supplement to prophylactic HPV vaccines.

## 3. Therapeutic HPV Vaccine

Currently, no therapeutic HPV vaccine has been approved for marketing worldwide, but clinical research and development is actively advancing. Based on the different vaccine technology platforms, the global research and development of therapeutic HPV vaccines are mainly divided into the following categories: nucleic acid vaccines (DNA vaccines, RNA vaccines), peptide vaccines, protein vaccines, vector vaccines (viral vector vaccines, bacterial vector vaccines), and cell-based vaccines. Each vaccine platform has its own advantages and disadvantages, and further details are shown in Table 1.

Therapeutic HPV vaccines are in different research stages, where most of them are in phase 1 or 2, and a few have entered phase 3, such as modified vaccinia Ankara E2 (MVA E2) [21], VGX-3100 (NCT03721978, NCT03721978), and ADXS-HPV (NCT02853604). The indications mainly focus on promoting the regression of CIN2/3 lesions and CC, with a few targeting the clearance of high-risk HPV infections and the regression of Vulvar Intraepithelial Neoplasia (VIN) and CIN 1, and a minority aimed at preventing recurrence after treatment for CIN and CC.

### 3.1. Mechanism of HPV Therapeutic Vaccines

Unlike prophylactic vaccines, therapeutic vaccines aim to activate or enhance the body’s cellular immunity to combat invading HPV or abnormal cells [22]. There are significant differences in composition and mechanisms of action between the two, as detailed in Table 2.

Antigens from viruses are potentially optimal targets for antigens in cancers linked to viral infections [23]. The expression of high-risk HPV oncoproteins E6 and E7 is a key factor in the malignant transformation of cervical epithelial cells and the maintenance of the oncogenic phenotype, so many current studies are targeting E6 and E7 for vaccine development [24]. There are also a few vaccines that target other early proteins, such as the MVA E2, which is a therapeutic HPV vaccine designed to target E2 alone [21]. There are also vaccines that combine other early proteins with E6 and E7 as targets, such as that described by Hancock et al. [25], who selected conserved regions from six early proteins, synthesizing a sequence of “5GHPV3” that includes common sequences of high-risk HPV 16, 18, 31, 52, and 58, and developed a multi-genotype therapeutic HPV vaccine. Currently, this vaccine is planned to undergo phase 1/2 clinical trials in female volunteers aged 25 to 55 (NCT04607850), with the trial set to be completed in January 2024. It is currently in the follow-up stage, and the results are eagerly anticipated. Some studies have shown that the incorporation of E2 and E5 into the vaccine design may trigger a stronger tumor-specific CD8+ T cell immune response compared to vaccines designed with only E6 and E7 as target antigens [26].

Traditional vaccines such as DNA vaccines, RNA vaccines, peptide vaccines, protein vaccines, and vector vaccines deliver the target antigens (E6, E7, E2, E5, etc.) to the body in different forms. First, the vaccine is delivered to antigen presenting cells (APCs), which then present the target antigenic epitopes on their surface through major histocompatibility complex class I (MHC I) and MHC II, respectively, to activate CD8+ cytotoxic T cells and CD4+ helper T cells. With the assistance of CD4+ helper T cells, the activated HPV antigen-specific CD8+ T cells recognize and kill cells infected with HPV and cancer cells. Under the action of CD4+ T cells, B cells are stimulated to produce neutralizing antibodies. Therefore, the purpose of traditional therapeutic HPV vaccines is to induce specific cellular immune responses, with humoral immunity as an auxiliary, in order to clear persistent HPV infections [22,23].

**Figure 1:**
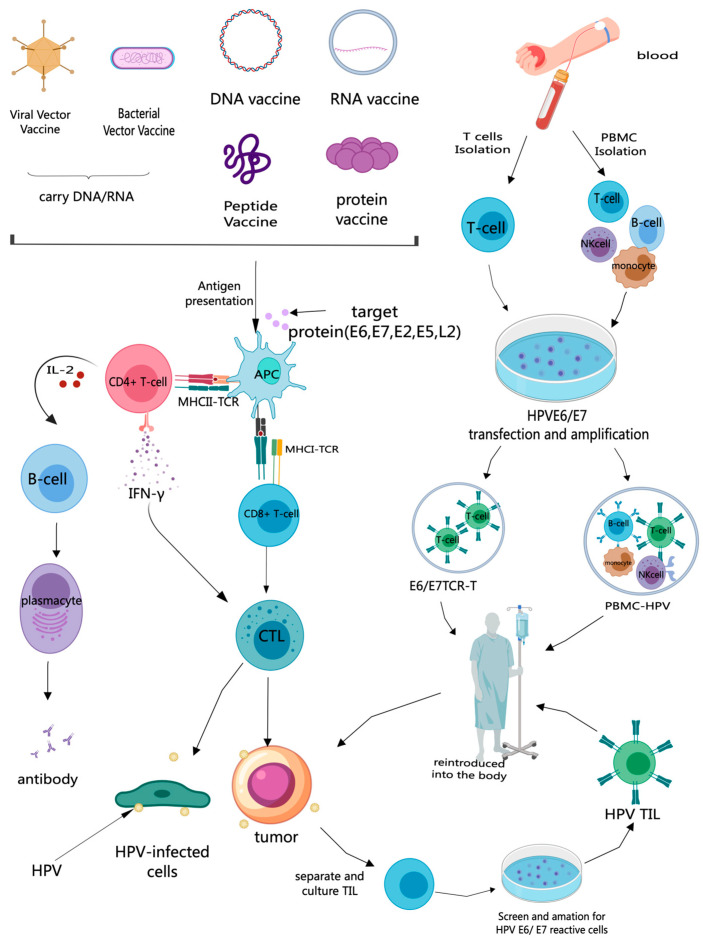
Mechanism of therapeutic HPV vaccines. Traditional vaccines such as DNA vaccines, RNA vaccines, peptide vaccines, protein vaccines, and vector vaccines can deliver target antigens to antigen-presenting cells (APCs), which then present the target antigen epitopes on their surface through major histocompatibility complex class I (MHC I) and MHC II. With the assistance of CD4+ helper T cells, activated HPV antigen-specific CD8+ T cells produce cytotoxic T lymphocytes (CTLs), which kill cells infected with HPV [22,23]. HPV Tumor Infiltrating Lymphocyte (HPV-TIL) vaccines [31] involve the process of separating and culturing TILs from the patient’s tumor tissue, and then screening lymphocytes that are reactive to HPV E6/E7 antigens for reinfusion back into the patient. Isolating T cells or other immune cells from the patient’s blood, transfecting and amplifying these cells ex vivo with HPV E6/E7, and preparing them into T cell receptor-engineered T cell (TCR-T) vaccines [27] and autologous peripheral blood mononuclear cell (PBMC) vaccines [28], followed by reinfusion back into the patient.

On the other hand, cell-based vaccines involve isolating T cells or other immune cells from the patient’s blood or tumor, ex vivo transduction with HPV E6/E7, preparation into HPV cell-based vaccines, and then reinfusion into the patient [27,28,29], such as the T cell receptor-engineered T cell (TCR-T) vaccine [27], dendritic cell (DC) vaccines [30], immunotherapeutic vaccines based on B cells and monocytes [29], HPV Tumor Infiltrating Lymphocyte (HPV-TIL) vaccines [31], and autologous peripheral blood mononuclear cell (PBMC) vaccines [28] (Figure 1).

### 3.2. Treatment of Persistent High-Risk HPV Infection and CIN

CIN is a precancerous condition, categorized into three stages (1, 2, and 3). It is estimated that CIN2-3 may persist or progress to cervical cancer after 10–20 years [32]. Untreated CIN2 may progress in 18% of cases [33]; untreated CIN3 has a 31% chance of advancing to invasive cancer within 30 years [32]. The treatment of CIN has not seen innovative improvements, and the primary treatment methods are surgery, including cold knife conization (CKC) and the loop electrosurgical excision procedure (LEEP). However, these surgeries have certain limitations and risks, such as pain, heavy bleeding, decreased fertility, and preterm labor [34,35]. Most importantly, HPV infection cannot be eradicated by currently available treatments. After conization, the HPV positivity rate among patients with high-grade CIN still ranges from 4.7% to 32.8%, and as many as 20.9% of patients develop CIN following their initial conization surgery [36]. So, it is crucial to develop non-surgical treatments for CIN. A recent meta-analysis has shown that therapeutic HPV vaccines are effective in the regression of CIN2 and CIN3 lesions without significant adverse reactions [37]. This meta-analysis included seven Randomized Controlled Trial (RCT) studies involving CIN2 and CIN3, with a total of 677 participants, using the regression of lesions to CIN1 or lower as the endpoint for efficacy. The results of four studies showed a significant difference in lesion regression between the vaccine group and the placebo group (combined *p*-value of 0.010). All studies reported good tolerance of the vaccine [37]. These vaccines are less invasive, easy to operate, and can avoid bleeding, infection and other complications associated with invasive surgical operations, making them a promising therapeutic approach.

The application of therapeutic HPV vaccines for CIN mainly focuses on three aspects: (i) to clear high-risk HPV infections and low-grade lesions (CIN1); (ii) to induce regression of VIN; and (iii) to induce regression of CIN2/3 (refer to Table 3 for further details).

#### 3.2.1. Clearance of High-Risk HPV Infection and Low-Grade Lesions (CIN1)

Therapeutic vaccine candidates targeting high-risk HPV infections and CIN1 are designed to clear specific high-risk HPV subtype infections and prevent HPV-related CIN2/3 and even cancer progression. Currently, there are relatively few such vaccines, and most have primary endpoints of HPV clearance rates or CIN1 regression rates. TA-CIN, a protein vaccine targeting HPV16, showed in a phase 2 clinical study (NCT01957878) that focused on HPV 16 and 18 infection in women with normal cytology or Atypical Squamous Cells of Undetermined Significance (ASCUS)/Low-grade Squamous Intraepithelial Lesion (LSIL) preliminary 12-month results indicating that the HPV clearance rate in the normal cytology and ASCUS group using TA-CIN was 58.7%, compared to 37.5% in the placebo group [38]. Another phase 2 clinical trial NCT03911076, which combined the DNA vaccine pNGVL4a Sig/E7(detox)/HSP70 and the protein vaccine TA-CIN, showed a 12-month result of a 64% HPV clearance rate (7/11) with TA-CIN [39], demonstrating the vaccine’s effect on HPV clearance.

Patients with CIN2/3 may undergo surgical treatment, but there is a lack of standard treatment methods for persistent high-risk HPV and CIN1. Currently, there is significant public demand for treatments in this area, yet clinical trials for therapeutic HPV vaccines are scarce. It is essential to develop more therapeutic HPV vaccines targeting persistent high-risk HPV and CIN1 to accelerate the global strategy for the elimination of CC.

#### 3.2.2. Induce Regression of VIN

VIN is a precancerous condition of vulvar cancer, and its incidence is showing a marked increasing trend [40]. A study on the viral vaccine TA-HPV, which targets the E6 and E7 of HPV 16 and 18, administered via intramuscular injection to patients with high-grade VIN, showed that after 24 weeks of treatment, 42% of patients experienced a partial regression in total lesion diameter [41]. The results of the phase 1 clinical trial NCT03180684 indicated that the DNA vaccine VGX-3100, which targets the E6 and E7 of HPV16 and HPV18, in combination with Imiquimod, led to a significant clinical regression in VIN in 63% of the patients after 6 months of treatment [42].

Currently, there are few therapeutic HPV vaccine candidates targeting VIN. When designing clinical trials for vaccines aimed at CIN, inclusion of the clearance rate of HPV infections at non-cervical sites should be considered (such as the anus, vulva, vagina, etc.) as a secondary clinical endpoint. For instance, a phase 3 clinical trial of a viral vaccine (MVA E2) recruited 1356 patients (including 1176 female and 180 male patients). They received a total of six intradermal injections of 1 × 10^7^ MVA E2 viral particles in specific areas such as the cervix, urethra, and vulva. A complete regression of lesions was observed in 89.3% of female patients, with 100% of vulvar lesions completely regressed, and no significant adverse reactions were reported [21].

#### 3.2.3. Induce Regression of CIN2/3

There are currently therapeutic HPV vaccine candidates targeting CIN2/CIN3, and some clinical trials focus solely on treating CIN3. More than a dozen clinical trials have reached phase 2 or phase 3. Almost all trials involve monotherapy and do not combine with other drugs. Some research works have set up placebo-controlled groups (NCT01022346, NCT02596243, NCT01304524, NCT03185013, NCT0372197), while others have not set up control groups (NCT00075569, NCT02529930, NCT02139267, NCT02411019, NCT06015854, NCT01116245). The primary endpoint for most therapeutic HPV vaccines treating CIN2 or CIN3 patients is regression of the lesion, defined as a return to CIN1 or complete regression of the lesion.

The clearance rates of HPV and CIN in the therapeutic HPV vaccine group were significantly higher than those in the placebo group. A phase 2 clinical trial NCT01022346 demonstrated significant efficacy of the viral vector vaccine TG4001, which targets HPV16 E6 and E7, in the treatment of CIN. The trial revealed a superior complete regression rate in the vaccine group over placebo for CIN 2/3 (24% vs. 10%, *p* < 0.05) and a marked increase for CIN 3 (21% vs. 0%, *p* < 0.01) [43]. VGX-3100 is a DNA vaccine targeting the E6 and E7 of HPV16 and 18. Research on it has been conducted over 16 years, since its phase 1 clinical trial began in May 2008 and continued to April 2024: in March 2021, the results of the phase 3 clinical trial REVEAL1 (NCT03185013) showed that among the 193 CIN2/3 subjects counted, the proportion of subjects with a complete regression of lesions and HPV virus clearance in the vaccine group was 23.7% (31/131), while in the placebo group it was 11.3% (7/62) [44]. In March 2023, the results of the phase 3 clinical trial REVEAL2 (NCT03721978) showed that the proportion of subjects with a regression of lesions and virus clearance in the vaccine group was 27.6% (37/134), while in the placebo group it was 8.7% (6/69) [45].

The clinical trial results presented in Table 3 demonstrate the outstanding efficacy of therapeutic HPV vaccines in clearing HPV infections and CIN within weeks to months. Some clinical trials have shown that the long-term clearance effects of therapeutic HPV vaccines are higher than those of the control groups treated with surgery and physical/pharmacological therapies [21]. Or they are comparable to the effects of the surgery group [46]. For instance, in a phase 3 clinical trial, among the control group treated with surgery and physical/pharmacological therapies, although the short-term elimination of CIN was successfully completed, 89% of patients experienced a recurrence of new lesions within 24 months after treatment. In the group treated with the therapeutic HPV vaccine alone, only 3.54% of patients had a recurrence of new lesions [21]. In a phase 2 RCT study, 91% (32/35) of women treated with VGX-3100 saw a regression of cervical HSIL within 6 months after the completion of the study treatment, avoiding surgery, and no HPV16/18 was detected 18 months after treatment completion. These results are comparable to those of patients who received a placebo and then underwent surgery [46]. However, the follow-up time in clinical trials was typically limited, with the longest follow-up period having been 2.5 years [43]. Therefore, the long-term clearance effect data currently observed are mostly based on follow-up results within 2.5 years. Further clinical trials and long-term follow-ups are needed to determine the long-term efficacy of the vaccine. Post-marketing real-world studies are also crucial for assessing the long-term effects of therapeutic HPV vaccines.

The aforementioned completed studies have demonstrated that after vaccination with therapeutic HPV vaccines, CIN 2/3 can regress to low-grade or non-cancerous precancerous conditions, significantly improving the efficacy compared to natural regression. Therapeutic HPV vaccines may potentially serve as an alternative to surgical treatment for high-grade lesions. Therapeutic HPV vaccines are suitable as an alternative or adjunct treatment for women who test positive in screening, avoiding postoperative complications. Based on HPV detection results, targeted vaccination can be carried out to more precisely treat existing precancerous conditions and prevent their further progression into cervical cancer [20].

All vaccines are multi-dose products, with the most common being a three-dose regimen that requires administration over several months with set intervals. The WHO, in its recommendations for the development and implementation strategies of therapeutic HPV vaccines, mentions that while the ideal vaccination schedule would be a single dose, multiple doses are also acceptable, and the immune response needs to be optimized. Ideally, vaccination should be performed when HPV test results are positive, preferably on the same day as the test. Regarding follow-up, the strategy mentioned by WHO is to align with existing cervical cancer screening and treatment programs [20].

**Table 3:** Clinical trials of therapeutic HPV vaccine in persistent high-risk HPV infection and CIN and VIN.

Indications	Combined Treatment	Vaccine Platform	Vaccine Name	HPV Types	Clinical Trial	Research Phase	Current State	Year	Number of Patients	Efficacy	Reference
Normal cytology or ASCUS/LSIL	no	protein vaccine	TA-CIN	HPV16-E6/E7/L2	NCT01957878	Phase 2, RCT, placebo-controlled	Completed	2013–2016	239	At 12 months, the HPV clearance rate was 58.7% compared to 37.5% in the placebo group.	[38]
ASC-US, ASC-H or LSIL	no	protein vaccine and DNA vaccine	TA-CIN and pNGVL4a Sig/E7(detox)/HSP70	HPV16-E6/E7/L2	NCT03911076	Phase 2, single-arm	Terminated	2019–2022	16	At 12 month, 64% (7/11) were HPV16-negative. In total, 36% (4/11) had normal cytology.	[39]
ASCUS or LSIL	no	protein vaccine	SGN-00101	HPV16-E7	NCT00091130	Phase 2, RCT, placebo-controlled	Completed	2004–2007	139	Unknown.	[47]
Persistent ASC-US/LSI L (>6 month period)	no	protein vaccine and DNA vaccine	TA-CIN and pNGVL4aCRTE6E7L2	HPV16-E6/E7/L2	NCT03913117	Phase 1, single-arm	Recruiting	Started in 2021, until now	Target: 30	Recruiting.	[48]
CIN2/3	no	DNA vaccine	VGX-3100	HPV16/18-E6/E7	NCT03721978 REVEAL 2	Phase 3, RCT, placebo-controlled	Completed	2019–2022	203	At week 36, lesion regression with viral clearance: vaccinated group = 27.6% (37/134), placebo group = 8.7% (6/69).	[45]
CIN2/3	no	bacterial vector vaccine	ADXS11-001	HPV 16-E7	NCT01116245	Phase 2, RCT, placebo-controlled	Terminated	2010–2016	81	Unknown.	[49]
CIN 2/3	no	viral vector vaccine	TG4001	HPV 16- E6/E7	NCT01022346	Phase 2, RCT, placebo-controlled	Completed	2009–2013	206	Follow-up results after 2.5-years: CIN two-thirds complete regression rate: vaccine group = 24%, placebo group = 10%; CIN 3 complete regression rate: vaccine group = 21%, placebo group = 0%.	[43]
CIN2/3	no	DNA vaccine	VB10.16	HPV16-E6/E7	NCT02529930	Phase 1/phase 2, single-arm	Completed	2015–2019	34	At 12 months, HPV16 clearance = 47% (8/17), lesions became smaller = 94% (16/17), lesion regression to CIN1 or normal = 59% (10/17).	[50]
CIN1/2/3	no	viral vector vaccine	MVA E2	HPV-E2	no	Phase 3, non-randomized, two-arm	Completed	Published in 2014	1176	Experimental group: complete lesion regression = 89% (1051/1184), lesion regression to CIN 1 = 2.4% (28/1184). Control group (surgery and physical/pharmacological treatments): 89% had new lesion recurrence within 24 months.	[21]
CIN 3	no	protein vaccine	SGN-00101(HspE7)	HPV16-E7	NCT00075569	Phase 2, non-placebo-controlled	Completed	2004–2005	64	The results of the 1–2 month follow-up: lesion regression to CIN1 or normal = 22.5% (13/58), >50% regression of lesion size = 55% (32/58), stable disease = 19% (11/58)	[51]
HSIL	no	peptide vaccine	PepCan	HPV16-E6	NCT02481414	Phase 2, RCT	Completed	2015–2022	81	At 15 months, pathological regression rate (PepCan group) = 30.8%, pathological regression rate (Candin group) = 47.6%.	[52,53]
CIN 3	no	DNA vaccine	GX-188E	HPV16/18-E6/E7	NCT02139267	Phase 2, non-placebo-controlled	Completed	2014–2016	72	At 20 weeks, histopathologic regression rate = 67% (35/52).	[54]
CIN 3	no	DNA vaccine	GX-188E	HPV16/18-E6/E7	NCT02411019	Phase 2, non-placebo-controlled	Unknown	Started in 2015	67	Unknown.	[55]
CIN 3	no	DNA vaccine	GX-188E	HPV16/18-E6/E7	NCT02596243	Phase 2, RCT, placebo-controlled	Unknown	Started in 2015	124	Unknown.	[56]
CIN3	GX-I7 or Imiquimod	DNA vaccine	GX-188E	HPV16/18-E6/E7	NCT03206138	unknown, non-placebo-controlled	Unknown	Started in 2017	50	Unknown.	[57]
CIN3	no	viral vector vaccine	Vvax001	HPV16-E6/E7	NCT06015854	Phase 2, single-arm	Recruiting	Starting in 2021, until now	Target: 18	Recruiting.	[58]
HSIL	no	DNA vaccine	VGX-3100	HPV16/18-E6/E7	NCT01304524	Phase 2, RCT, placebo-controlled	Completed	2011–2015	107	Histopathological regression at 36 weeks: VGX-3100 group = 49.5% (53/107), placebo group = 30.6% (11/36). At 18 months: cytological improvement—vaccine group (no surgery) = 100% (32/32), placebo group (underwent surgery) = 100% (45/45), HPV clearance rate. Vaccine group = 91%, placebo group = 88%.	[46,59]
HSIL	no	DNA vaccine	VGX-3100	HPV16/18-E6/E7	NCT03185013	Phase 3, RCT, placebo-controlled	Completed	2017–2021	201	HSIL histopathologic regression and HPV-16/18 clearance at week 36: treatment group = 23.7% (31/131), placebo group = 11.3% (7/62)	[44]
VIN 2/3	Imiquimod	DNA vaccine	VGX-3100	HPV16/18-E6/E7	NCT03180684	Phase 2, non-placebo-controlled	Completed	2017–2020	33	After six months of treatment, 63% (12/19) experienced ≥25% reduction in lesion area.	[42]

HPV, human papillomavirus; CIN, cervical intraepithelial neoplasia; VIN, Vulvar Intraepithelial Neoplasia; ASCUS, Atypical Squamous Cells of Undetermined Significance; LSIL, Low-grade Squamous Intraepithelial Lesion; HSIL, high-grade squamous intraepithelial lesion; ASC-H, Atypical squamous cells: cannot exclude high-grade squamous intraepithelial lesion; RCT, Randomized Controlled Trial.

### 3.3. Treatment of Cervical Cancer

Therapeutic HPV vaccines are being explored across multiple clinical trials for CC treatment, showing potential in first-line therapy for primary disease, and second-line for recurrent cases. The therapeutic HPV vaccines are primarily applied in CC in three main ways: (i) used alone as a second-line treatment for recurrent, metastatic, and refractory CC; (ii) in combination with traditional chemotherapy or chemoradiotherapy for first-line and second-line treatment of CC; and (iii) in combination with other immunomodulatory agents for second-line treatment of advanced, recurrent, metastatic, and refractory CC (refer to Table 4 for further details).

#### 3.3.1. Second-Line Treatment for Recurrent, Metastatic, Refractory CC Alone

Solo use of therapeutic HPV vaccines for the treatment of CC has been relatively limited, with most trials being exploratory and there not yet being large-scale RCT studies. Although the existing clinical trial results are sparse, there is a trend indicating that CC patients can benefit from HPV vaccines. In a phase 2 trial, the HPV16 E7-targeting viral vaccine ADXS11-001 achieved a 12-month survival rate of 38% in patients with recurrent metastatic CC, surpassing the historical rate of 25% [60]. In the phase 2a trial NCT02866006, BVAC-C, an immunotherapy vaccine leveraging B cells and monocytes, demonstrated an objective response rate (ORR) of 19.2% and a disease control rate (DCR) of 53.8% in patients with recurrent HPV 16- or 18-positive CC, with a median overall survival (OS) of 17.7 months, indicating a positive safety and efficacy profile [29]. A phase 1 clinical trial, NCT02858310, utilizing the TCR-T vaccine targeting HPV-16 E7, achieved objective responses in 6 out of 12 metastatic epithelial cancer patients, notably benefiting 4 patients previously resistant to anti-PD-1 therapy [61]. SQZ-PBMC-HPV is a PBMC cell-based vaccine targeting the HPV16-E6 and E7. In a phase 1 trial (NCT04084951) for treating patients with HPV16+ metastatic solid tumors, the total disease control rate was 33.3% [28].

However, high tumor load, multi-drug resistance, and immunodeficiency are frequently encountered in patients with advanced cancer, especially those receiving multiple lines of therapy. Several combination strategies can enhance anti-tumor immunity [23]. Therefore, the combination of therapeutic HPV vaccines with other therapies may prolong the survival of patients.

#### 3.3.2. Combination with Chemotherapy or Chemoradiotherapy for First- and Second-Line Treatment of CC

Theoretically, chemotherapy can change the traits of tumor cells, thereby improving immunotherapy results [62]. One example is the effect of cisplatin on increasing the number of CD11c+DCs at the tumor site, making it a favorable location for therapeutic HPV vaccines to trigger tumor-specific immune responses [63]. Currently, in clinical trials, chemotherapeutic agents combined with therapeutic HPV vaccines include cisplatin [64], carboplatin, paclitaxel [65], topotecan [29,66], cyclophosphamide, and fludarabine [67].

In fact, some clinical trial results have shown that the therapeutic vaccine alone is as effective as when used in combination with chemotherapy [64], and even more effective in some cases, with fewer adverse reactions [29]. For instance, the median progression-free survival (6.10 months vs. 6.08 months) and overall response rate (17.1% vs. 14.7%) of ADXS11-001 used alone are comparable to those used in combination with cisplatin, and it has fewer adverse reactions [64]. BVAC-C, a cell-based vaccine based on B cells and monocytes, shows better results when used alone compared to its use in combination with chemotherapy (topotecan), with ORR of 20.0% and 0%, and DCR of 57.1% and 14.3%, respectively [29]. The reason for this situation may be that the mechanisms of action of some chemotherapy drugs are mutually exclusive with vaccines [29,66]; hence, it is necessary to explore the combination of chemotherapy drugs and vaccines.

Several studies have explored the optimal timing and dosage of vaccine administration in combination with chemotherapy to achieve the best therapeutic effects. A phase 1/2 clinical trial (NCT02128126) included 77 patients with HPV 16+ CC that had progressed, metastasized, or recurred. All patients were administered both chemotherapy and the peptide vaccine ISA101. The patients received four different vaccine dosages: 20 μg, 40 μg, 100 μg, or 300 μg. The results showed that chemotherapy effectively reduced the number of suppressive myeloid cells. Furthermore, the vaccine induced a robust T cell response at different dosage levels. Therapeutic effects also mirrored the immune responses. In 43% of the patients, tumor regression was noted. In total, 11 out of the 14 patients demonstrated a robust immune response triggered by the vaccine, with 3 of them having advanced stage IVa/IVb disease and surviving an average of over 3 years [68]. A different study involving patients with advanced CC found that the ideal timing to begin administering the therapeutic HPV vaccine ISA101 was two weeks following the second round of carboplatin/paclitaxel treatment. By this stage, the excessively high levels of immunosuppressive myeloid cells in the body had decreased to levels comparable to those seen in healthy individuals [65].

Radiotherapy not only destroys tumor cells directly, but also triggers immunogenic cell death, which can be recognized by immune cells. It also enhances the activation of dendritic cells and facilitates efficient antigen presentation, leading to a transformation of the immunosuppressive tumor microenvironment that boosts anti-tumor immunity [69]. During the 2022 annual meeting of The Society for Immunotherapy of Cancer (SITC), findings from the phase 2 IMMUNOCERVtrial (NCT04580771) revealed that the combination of therapeutic HPV vaccine PDS0101 with chemoradiotherapy resulted in a 100% ORR in nine patients diagnosed with high-risk locally advanced CC. The treatment led to substantial tumor regression of over 60%, with eight patients achieving complete response (CR) and one experiencing partial response (PR). Out of the nine patients who finished the treatment, eight were still alive at the data cutoff. At the interim evaluation using Magnetic Resonance Imaging (MRI), a tumor regression exceeding 60% was observed [70]. The AIM2CERV phase 3 clinical trial (NCT02853604) is assessing the efficacy of ADXS11-001 as an adjuvant immunotherapy in patients with high-risk HPV-related locally advanced CC following chemotherapy or radiotherapy.

#### 3.3.3. Utilization in Conjunction with Other Immunological Agents for the Second-Line Management of Advanced, Recurrent, Metastatic, and Refractory CC

Immune checkpoint inhibitors (ICIs) have shown promise in treating CC, but ICIs’ potential in treating cancer is hampered by limited T cell responses [71]. Cancer vaccines are advancing the transformation of “cold” tumors into “hot” ones by stimulating T cell responses, and their combination with ICIs like pembrolizumab is showing enhanced survival rates and objective response rates, warranting further clinical investigation [23].

Some clinical trials have demonstrated that the combination of therapeutic HPV vaccines with PD-1/PD-L1 inhibitors has better clinical efficacy than monotherapy with immunotherapy alone. Pembrolizumab has received approval for treating recurrent or metastatic CC, demonstrating an ORR of 14.3% [72]. The phase 2 trial NCT03444376 combining the HPV16/18-targeting DNA vaccine GX-188E with pembrolizumab reported a 35.0% ORR and a median OS of 23.8 months, displaying robust anti-tumor activity regardless of PD-L1 status [73].

Multiple clinical studies have integrated therapeutic HPV vaccines with PD1/PD-L1 inhibitors, demonstrating clinical benefits from the combination irrespective of PD-L1 expression. Patients with positive PD-L1 expression showed more pronounced benefits. The ORR of atezolizumab monotherapy for advanced cervical cancer is 14.8% [74]. The phase 2 trial NCT04405349 of VB10.16 DNA vaccine with atezolizumab as a second-line treatment for HPV16-positive advanced CC achieved an ORR of 21% and a DCR of 64%. Significant anti-tumor effects were noted in both PD-L1+ (ORR 27%, DCR 77%) and PD-L1- patients (ORR 17%, DCR 58%), suggesting broad clinical promise [75]. This year, a follow-up study (NCT06099418) has been launched to enroll advanced HPV16+patients with PD-L1+CC who have become resistant to pembrolizumab with or without bevacizumab. In the treatment of recurrent cervical cancer, 16.4% of patients in the cemiplimab group experienced objective responses, compared to 6.3% in the chemotherapy group. Furthermore, 18% of patients treated with cemiplimab had PD-L1 expression of 1% or greater [76]. The phase 2 trial NCT04646005, which combined the ISA101b vaccine with cemiplimab, reported an ORR of 16.8%, with a higher ORR of 22.4% in patients with a PD-L1 expression of 1% or greater. This indicates that for patients with a PD-L1 expression of 1% or greater, the combination therapy of cemiplimab and the vaccine showed better clinical efficacy compared to monotherapy with either immunotherapy or chemotherapy alone [77].

The phase 2 DURBAC study NCT04800978, combining durvalumab with the BVAC-C vaccine, reported a 6-month PFS rate of 50% and an ORR of 38% in patients with recurrent or advanced HPV 16/18+ CC, highlighting the potential of this immunotherapy combination [78] and demonstrating the potential of combination therapy with cell-based vaccines and PD-L1 inhibitors for the treatment of recurrent CC.

The results of the aforementioned studies indicate that the combination of ICIs with therapeutic HPV vaccines has a high clinical response rate and manageable safety, effectively achieving the “cold–hot” transformation of tumors, activating the body’s anti-tumor immunity, and achieving a “1 + 1 > 2” effect.

Overall, monotherapy with therapeutic HPV vaccines for the treatment of CC has limited indications and efficacy. The combination of vaccines with chemoradiotherapy and immunotherapy drugs can elicit more robust immune responses and better therapeutic outcomes. Compared to conventional adjuvant treatment methods such as chemotherapy and radiotherapy, therapeutic HPV vaccines do not directly kill tumor cells and rapidly dividing normal cells in the body, thus avoiding severe side effects. Therefore, therapeutic HPV vaccines are a gentler and a more targeted treatment method for CC.

**Table 4:** Clinical trials of therapeutic HPV vaccine in cervical cancer.

Indications	Combined Treatment	Vaccine Platform	Vaccine Name	HPV Types	Clinical Trial	Research Phase	CURRENT STATE	Year	Number of Patients	Efficacy	Reference
Early CC	Surgery	Viral vector vaccine	TA-HPV	HPV16/18-E6/E7	NCT00002916	Phase 2, two-arm, non-randomized	Completed	Started in 2017	Target 44	Unknown	[79]
Stage IB3-IVA CC	Radiotherapy	Peptide vaccine	PDS0101	HPV 16-E6/E7	NCT04580771	Phase 2, single-arm	Recruitment completed	2022	17	ORR = 100% (8 CR, 1 PR)	[70]
Stage IIB-IVA CC	No	Cell-based vaccine	E7 T-Cell Receptor (TCR-T)	HPV16-E7	NCT04476251	Phase 1, single-arm	Terminated	2021	1	One participant was enrolled but not treated	[80]
HPV16+ R/M solid tumors with HLA-A*02+	No	Cell-based vaccine	SQZ-PBMC-HPV	HPV16-E6/E7	NCT04084951	Phase 1, single-arm	Completed	2020–2023	18	Overall DCR 33.3%	[28]
HPV-associated R/M solid tumors	Combined or not with M7824 (MSB0011359C) (dual PD-L1 and TGF- beta inhibitor)	Viral vector vaccine	PRGN-2009	HPV 16/18-E6/E7	NCT04432597	Phase 1/phase 2, multi-armed, non-randomized	Recruitment completed	Starting in 2020, until now	39	Phase 1 results: ORR = 30.0%, OS (PRGN-2009) = 7.4 months, OS (combination) = 12.5 months	[81]
Pembrolizumab-resistant R/M CC	Pembrolizumab (PD-1 inhibitor)	Viral vector vaccine	PRGN-2009	HPV 16/18 -E6/E7	NCT06157151	Phase 2, RCT, placebo-controlled	Recruiting	Starting in 2024, until now	Target 46	Recruiting	[82]
HPV-associated cancers	Atezolizumab (PD-L1 inhibitor)	Viral vector vaccine	MG1-E6E7	HPV16/18-E6/E7	NCT03618953	Phase 1, non-placebo-controlled	Terminated	2018–2021	8	Terminated	[83]
HPV 16+ cancer with HLA-A*02+	Cyclophosphamide	Peptide vaccine	DPX-E7	HPV16-E7	NCT02865135	Phase 1/phase 2, single-arm	Completed	2017–2023	11	7 out of 11 PD, 3 SD.	[67]
Metastatic HPV+cancers	No	Cell-based vaccine	E7 TCR-T	HPV16-E7	NCT02858310	Phase 1/phase 2, single-arm	Phase 1 completed, phase 2 recruiting	Starting in 2018, until now	12,(5 cervix,1 vulva)	Objective clinical responses: 6/12 patients, including 4 of 8 patients with anti-PD-1 refractory disease	[61]
IB1-IVA CC or persistent/ recurrent CC	After chemoradiation	DNA vaccine	NO-3112 (MEDI0457)	HPV 16/18- E6/E7	NCT02172911	Phase 1, single-arm	Completed	2014–2020	10	Anti-HPV antibody responses: 6/10 patients, IFNγ-producing T cell responses: 6/10 patients, treatment-related adverse events: all grade 1	[84]
R/M HPV-16+ cancers	Avelumab (PD-L1 inhibitor)	Viral vector vaccine	TG4001	HPV16-E6/E7	NCT03260023	Phase 1/phase 2, single-arm	Recruitment completed	Starting in 2017, until now	43(18 cervix)	ORR = 22% (1 CR, 7 PR), DCR at 12 weeks = 42% (15/36), PFS = 2.8 months, OS = 11.0 months	[85]
Recurrent CC with disease progression after first-line chemotherapy	Cemiplimab (PD-1 inhibitor)	Peptide vaccine	ISA101b	HPV16-E6/E7	NCT04646005	Phase 2, single-arm	Recruitment completed	Starting in 2021, until now	113	ORR = 16.8%, ORR (PD-L1 <1%) = 12.5%, ORR (PD-L1 ≥1%) = 22.4%, OS = 13.3 months, PFS = 3.0 months	[77]
HPV-16 + incurable solid tumors	Nivolumab(PD-1 inhibitor)	Peptide vaccine	ISA101	HPV16-E6/E7	NCT02426892	Phase 2, single-arm	Completed	2015–2021	33	Median DOR = 11.2 months, PFS = 2.66 months, OS = 15.3 months, 2-year OS rate = 33%	[86]
HPV16+ R/M CC	Pegylated interferon alpha (IFN α)	Peptide vaccine	ISA101/ISA101b	HPV16-E6/E7	NCT02128126 CervISA	Phase 1/phase 2, single-arm	Completed	2013–2018	93	Tumor regressions = 43%, stable disease = 43% (62/72 patients), OS (low response) = 11.2 months, OS (strong response) = 16.8 months	[68]
CC refractory after platinum-based first-line chemotherapy	Durvalumab (PD-L1 inhibitor)	Cell-based vaccine	BVAC-C	HPV16/18-E6/E7	NCT04800978	Phase 2, single-arm	In progress	Starting in 2021, until now	Target 37	Interim analysis: 6-month PFS rate = 50%, ORR = 38%, CR = 14%, PR = 24%, DOR = 13.3 months, median PFS = 8.7 months	[78]
Advanced or recurrent non-resectable CC with failure of standard therapy	Atezolizumab (PD-L1 inhibitor)	DNA vaccine	VB10.16	HPV16- E6/7	NCT04405349	Phase 2, single-arm	Completed	2020–2023	52	Interim results (39 patients, median follow-up 6 months): ORR = 21% (2 CR, 6 PR), DCR = 64%, ORR (PD-L1+) = 27%, DCR (PD-L1+) = 77%, ORR (PD-L1-) = 17%, DCR (PD-L1-) = 58%	[75]
R/M HPV16+, PD-L1+ CC	Combined or not with atezolizumab (PD-L1 inhibitor)	DNA vaccine	VB10.16	HPV16- E6/E7	NCT06099418	Phase 2, single-arm	Withdrawn	Starting in 2024, until now	Target 130	Withdrawn	[87]
Advanced, non-resectable CC	Pembrolizumab (PD-1 inhibitor)	DNA vaccine	GX-188E	HPV16/18-E6/E7	NCT03444376	Phase 1/phase 2, single-arm	Completed	2018–2023	65	24-week ORR = 35.0%, CR = 8.3%, PR = 26.7%, median PFS = 4.4 months, median OS = 23.8 months	[73]
HPV16+ cancer	No	RNA vaccine	HARE-40 (HPV Anti-CD40 RNA Vaccine)	HPV16-E7	NCT03418480	Phase 1/phase 2, non-placebo-controlled	Completed	2017–2024	32	Not yet reported	[88]
R/M CC with failure of conventional therapy	No	Bacterial vector vaccine	ADXS11-001	HPV 16-E7	NCT02164461	Phase 1, single-arm	Completed	2015–2017	12	The increased dosage of ADXS11-001 demonstrated good tolerability, establishing an RP2D at 1 × 10^10^ CFU	[89]
Platinum- refractory CC	No	Bacterial vector vaccine	ADXS-HPV	HPV 16-E7	NCT01266460	Phase 2, single-arm	Completed	2011–2018	50	12-month OS = 38% (*n* = 19), median OS = 6.1 months, median PFS = 2.8 months	[60]
Advanced CC after first-line treatment	Combined or not with cisplatin	Bacterial vector vaccine	ADXS11-001	HPV 16-E7	CTRI/2010/091/001232	Phase 2, RCT, placebo-controlled	Completed	2018	109	Median OS (ADXS11-001) = 8.28 months (95% CI: 5.85–10.5), median OS (ADXS11-001 + cisplatin) = 8.78 months, median PFS (both groups) = 6.09 months, ORR (ADXS11-001) = 17.1%, ORR (ADXS11-001 + cisplatin) = 14.7%	[64]
Checkpoint inhibitor (CPI) primary and CPI-refractory advanced HPV-positive cancers	M9241, bintrafusp alfa	Peptide vaccine	PDS0101	HPV 16-E6/E7	NCT04287868	Phase 2, single-arm	In progress	Starting in 2022, until now	29	Median OS (PDS0101 + M9241 + bintrafusp alfa) = 21 months (*n* = 29), ORR (CPI-refractory) = 63%, ORR (CPI-primary) = 88%	[90]
Recurrent/Metastatic HPV-associated cancers	Durvalumab (PD-L1 inhibitor)	DNA vaccine	INO-3112 (MEDI0457)	HPV 16/18-E6/E7	NCT03439085	Phase 2, single-arm	Completed	2018–2022	21 (12 cervix)	ORR = 21%, DCR = 37%, DOR = 21.8 months, PFS = 4.6 months, OS = 17.7 months, grades 3–4 AE = 23%	[91]
Metastatic or locally advanced refractory or recurrent CC	No	Cell-based vaccine	HPV-TILs	HPV 16/18-E6/E7	NCT01585428	Phase 2, single-arm	Completed	2012–2016	29	Objective tumor responses = 3/9 patients (2 CR, 1 PR), duration of CR = 22 months and 15 months	[31]

HPV, human papillomavirus; CC, cervical cancer; CR, complete response; PR, partial response; TCR-T, T cell receptor-engineered T cell; R/M, recurrent or metastatic; DCR, disease control rate; OS, overall survival; PD, Progressive Disease; SD, stable disease; PFS, progression-free survival; ORR, objective response rate; CI, Confidence Interval; CPI, checkpoint inhibitor; AE, adverse events; PD-1, Programmed Cell Death Protein 1; PD-L1, Programmed Cell Death Ligand 1; IFNγ, interferon gamma; RCT, Randomized Controlled Trial.

### 3.4. Prophylaxis Following Completion of Treatment

The application of therapeutic HPV vaccines for prophylaxis after completion of treatment mainly focuses on two aspects: (i) prophylaxis after local surgery for CIN; and (ii) prophylaxis following the completion of cervical cancer treatment. (Refer to Table 5 for further details.)

#### 3.4.1. Prophylaxis After Local Surgery for CIN

Vvax001 is a viral vector vaccine targeting the E6 and E7 of HPV16 [92], a phase 1 clinical trial (NCT03141463) enrolled 12 patients with CIN 2/3 who had completed local surgical treatment. The results showed that the Vvax001 vaccine was immunologically safe and well-tolerated. Vvax001 induced CD4+ and CD8+ T cell responses against the E6 and E7 antigens, and Vvax001 induced a strong HPV16 E6 and E7 specific IFN-γ response. These data strongly support the use of Vvax001 as a therapeutic HPV vaccine that can generate sustained cellular responses after treatment completion, thereby preventing cervical virus infection and CIN recurrence more effectively in the long term, and warrant further clinical evaluation [93].

After surgical removal of the lesion, the antibodies induced by the HPV prophylactic vaccine can prevent new infections caused by different HPV types and reinfections by the same HPV type [14]. The administration of therapeutic vaccines can treat the same HPV infection and prevent the recurrence of CIN. The purposes of administering the two types of vaccines after local surgery are different, and whether they can be administered simultaneously is open to discussion.

#### 3.4.2. Prophylaxis Following the Completion of Cervical Cancer Treatment

A phase 1 study included 10 postoperative cervical cancer patients who received an HPV DC vaccine. All patients developed CD4+ T cell and antibody responses against the DC vaccine, and immune responses specific to E7 in CD8+ T cells were increased in eight patients [30]. Another phase 1 clinical study used the protein vaccine TA-CIN, targeting the E6/E7/L2 proteins of HPV16 (NCT02405221), to treat patients with stage IB1-IV cervical cancer related to HPV16 who completed definitive treatment within 12 months, with the aim of clearing HPV infection and preventing recurrence. This study is still ongoing.

**Table 5:** Clinical trials of therapeutic HPV vaccine in post-treatment prophylaxis.

Indications	Combined Treatment	Vaccine Platform	Vaccine Name	HPV Types	Clinical Trial	Research Phase	Current State	Year	Number of Patients	Efficacy	Reference
A history of CIN 2.3	no	Viral vector vaccine	Vvax001	HPV16-E6/E7	NCT03141463	Phase 1	Completed	2017–2017	12	Vvax001 is immunologically safe and well tolerated, Vvax001 causes CD4+ and CD8+ T cell response against E6 and E7 antigens, and Vvax001 induces strong HPV16 E6- and E7-specific IFN-γ responses.	[93]
Stage IB or IIA CC after undergoing radical surgery	no	Cell-based vaccine	HPV DC vaccine	HPV16/18-E7	no	Phase 1	Completed	2004–2006	10	All patients developed CD4(+) T cell and antibody responses to DC vaccination, and 8 out of 10 patients demonstrated levels of E7-specific CD8(+) T cell counts after immunization, which were increased compared to prevaccination baseline levels.	[30]
IB1-IV CC having completed treatment within 12 months	no	Protein vaccine	TA-CIN	HPV16-E6/E7/L2	NCT02405221	Phase 1	Recruitment completed	2019–2024	14	Not yet reported.	[94]

HPV, human papillomavirus; CIN, cervical intraepithelial neoplasia; IFN-γ, interferon gamma; CC, cervical cancer; DC, dendritic cell.

## 4. Characteristics and Limitations of Therapeutic HPV Vaccine Development

The current development of therapeutic HPV vaccines is characterized by the following: (i) A variety of platform vaccines are advancing in parallel, such as peptide, protein, DNA, RNA, and bacterial and viral vector vaccines. Each type of vaccine has a different mechanism of action, and all have their strengths and weaknesses, as shown in Table 1. (ii) New forms of vaccines are gradually evolving, such as mRNA vaccines and cell-based vaccines [27,28,29]. Preclinical experiments have shown that a single dose of HPV mRNA vaccine can induce a strong CD8+ T cell response, exerting a powerful anti-tumor effect, which is superior to HPV protein vaccines and HPV DNA vaccines [95]. A variety of cell-based vaccines, such as TCR-T [27], DC vaccines [30], immunotherapeutic vaccines based on B cells and monocytes [29], HPV-TIL vaccines [31]. and autologous PBMC cell vaccines [28], are all in development. (iii) Diversified delivery methods: In clinical studies, extragastrointestinal administration (including subcutaneous and intramuscular injection) is the most common vaccination route, and electroporation is a common delivery method for DNA vaccines. Some studies directly administer vaccines within the lesion to treat CIN [96] or inject within the cervical cancer tumor. New delivery methods are also being explored, such as lipid nanoparticle LNP delivery systems [97]. (iv) Combined therapy has become a trend: therapeutic HPV vaccines can also be used in conjunction with traditional treatment methods (such as surgery, radiotherapy, chemotherapy) and can have a synergistic effect when used with immunotherapy, and even two therapeutic HPV vaccines can be used in combination to improve treatment effects.

The therapeutic HPV vaccine development faces several challenges that need to be addressed: (i) Currently, therapeutic vaccines mainly target the E6 and E7 antigen genes of HPV, with less research on other potential antigen genes, hence the need to explore new targets. (ii) Some vaccines have shown poor immunogenicity, particularly peptide and protein vaccines. Optimal vaccine strategies are required to enhance their efficacy, such as vector optimization, novel vaccine design, delivery method enhancement, and the development of more potent adjuvants. (iii) Clinical trials for therapeutic HPV vaccines have design rigor issues. The absence of control groups in trials can diminish the credibility of results, and there are concerns regarding insufficient sample sizes. Compared to clinical trials for CIN, there are few two-arm studies involving patients with CC and with small sample sizes, lacking control groups, which can lack scientific rigor when compared with historical controls. There is a need for further RCTs to determine the efficacy of therapeutic HPV vaccines in cervical cancer. (iv) The therapeutic efficacy of T cells induced by vaccines may be compromised by various immune evasion and suppression mechanisms employed by tumors. It is essential to consider treatments that modulate the tumor microenvironment and to seek more combinations with radiochemotherapy and immunotherapy, exploring the right dosages and timing. (v) There is inadequate coverage of indications. For instance, fewer clinical trials focus on persistent high-risk HPV infections, CIN1, early-stage cervical cancer, and preventive treatments post-CIN and -CC treatments.

## 5. Conclusions and Perspectives

Prevention, screening, and treatment are key to the success of the global strategy to accelerate the elimination of cervical cancer. Therapeutic HPV vaccines can complement the deficiencies of prophylactic vaccines and have a broad range of applications. Numerous clinical trials on therapeutic HPV vaccines are underway, gradually demonstrating significant potential and value in treating HPV infections, CIN, and cervical cancer. To strive for the global goal of eliminating cervical cancer, further exploration and optimization of technologies and strategies are needed in the future to overcome limitations and improve the efficacy of therapeutic HPV vaccines. It is believed that with the efforts of all scientific researchers and medical workers, cervical cancer is expected to become the first cancer in human history to be completely eradicated.

## Figures and Tables

**Table 1:** Therapeutic HPV vaccines from different platforms and its advantages and disadvantages.

Vaccine Types	Representative Vaccines	Advantages	Disadvantages
DNA vaccine	- VB10.16	- Simple production.	- Low transfection efficiency.
	- GX-188E	- Repeatable vaccination.	- Low immunogenicity.
	- VGX-3100	- Low cost.	- Risk of autoimmune reactions.
	- NO-3112(MEDI0457)	- Good stability.	- Risk of integration with the human genome.
		- Long-lasting immune response.	
		-Multiple antigens can be delivered.	
RNA vaccine	- HARE-40	- No risk of integration into host genome.	- Poor stability.
			- Quick degradation.
		- Quick to develop, easy to modify. - High immunogenicity.	- May cause inflammatory reactions.

Peptide vaccine	- DPX-E7	- High specificity and safety	- Complex manufacture.
	- PepCan	- Low risk of autoimmunity.	- High cost.
	- ISA101/ISA101b	- Safe and tolerable.	- Limitation of MHC.
	- PDS0101		- Low immunogenicity.
Protein vaccine	- SGN-00101	- Safe and tolerable.	- Low immunogenicity.
	- TA-CIN	- No limitation of MHC.	- Easier to mediate humoral immunity.
Viral and bacterial vector vaccine	- PRGN-2009	- High transfection efficiency.	- Potentially pathogenic.
	- MG1-E6E7	- High immunogenicity.	- Immune response to vectors may exceed HPV antigens.
	-ADXS11-001	- Long immune response times.	
	- TG4001	- Intrinsic adjuvant properties.	- Antibodies against the vectors may be pre-existing.
	- Vvax001		
			- Neutralizing antibodies produced limit repeat treatments.
Cell-based vaccine	- HPV-TIL	- Strong immune stimulation.	- High cost.
	- E7 TCR T Cells	- Multi-form antigen loading.	- Highly demanding cell culture technology.
	- SQZ-PBMC-HPV		
	- BVAC-C		- Need for patient-specific customization.

MHC, major histocompatibility complex; HPV, human papillomavirus.

**Table 2:** Major differences between prophylactic and therapeutic HPV vaccines.

Feature	Prophylactic HPV Vaccine	Therapeutic HPV Vaccine
Indications	Prevention of new HPV infections	Clearance of existing HPV infections
Main Antigenic Epitopes	L1, L2 capsid proteins	E6, E7 oncogenic protein epitopes
Type of Immunity	Humoral immunity	Cellular immunity

HPV, human papillomavirus.

## Abbreviations

Cervical cancer (CC); Human Development Index (HDI); human papillomavirus (HPV); cervical intraepithelial neoplasia (CIN); World Health Organization (WHO); major histocompatibility complex (MHC); modified vaccinia Ankara E2 (MVA E2); antigen presenting cells (APCs); Deoxyribonucleic Acid (DNA); Ribonucleic Acid (RNA); Magnetic Resonance Imaging (MRI); The Society for Immunotherapy of Cancer (SITC); Vulvar Intraepithelial Neoplasia (VIN); cold knife conization (CKC); loop electrosurgical excision procedure (LEEP); Atypical Squamous Cells of Undetermined Significance (ASCUS); Low-grade Squamous Intraepithelial Lesion (LSIL); high-grade squamous intraepithelial lesion (HSIL); atypical squamous cells: cannot exclude high-grade squamous intraepithelial lesion (ASC-H); overall survival (OS); objective response rate (ORR); disease control rate (DCR); progression-free survival (PFS); immune checkpoint inhibitors (ICIs); Programmed Cell Death Protein 1 (PD-1); Programmed Cell Death Ligand 1 (PD-L1); interferon gamma (IFN-γ); Tumor Necrosis Factor Alpha (TNF-α); complete response (CR); partial response (PR); duration of response (DOR); treatment-emergent adverse events (TEAEs); T cell receptor-engineered T cell (TCR-T); dendritic cell (DC); Tumor Infiltrating Lymphocytes (HPV-TIL); peripheral blood mononuclear cells (PBMC); mRNA-encoded anti-CD40 (HARE-40); Confidence Interval(CI); checkpoint inhibitor (CPI); Randomized Controlled Trial (RCT).
